# Exopolysaccharide-producing bacteria enhanced Pb immobilization and influenced the microbiome composition in rhizosphere soil of pakchoi (*Brassica chinensis* L.)

**DOI:** 10.3389/fmicb.2023.1117312

**Published:** 2023-03-09

**Authors:** Ruiwen Cao, Yiling Zhang, Yuhao Ju, Wei Wang, Yanqiu Zhao, Nan Liu, Gangrui Zhang, Xingbao Wang, Xuesong Xie, Cunxi Dai, Yue Liu, Hongfei Yin, Kaiyuan Shi, Chenchen He, Weiyan Wang, Lingyu Zhao, Che Ok Jeon, Lujiang Hao

**Affiliations:** ^1^State Key Laboratory of Biobased Material and Green Papermaking, Qilu University of Technology (Shandong Academy of Sciences), Jinan, China; ^2^Department of Life Science, Chung-Ang University, Seoul, Republic of Korea

**Keywords:** marine bacteria, soil lead contamination, EPS-producing bacteria, Pb-immobilizing, pakchoi (*Brassica chinensis* L.), inter-rhizosphere microflora

## Abstract

Lead (Pb) contamination of planting soils is increasingly serious, leading to harmful effects on soil microflora and food safety. Exopolysaccharides (EPSs) are carbohydrate polymers produced and secreted by microorganisms, which are efficient biosorbent materials and has been widely used in wastewater treatment to remove heavy metals. However, the effects and underlying mechanism of EPS-producing marine bacteria on soil metal immobilization, plant growth and health remain unclear. The potential of *Pseudoalteromonas agarivorans* Hao 2018, a high EPS-producing marine bacterium, to produce EPS in soil filtrate, immobilize Pb, and inhibit its uptake by pakchoi (*Brassica chinensis* L.) was studied in this work. The effects of strain Hao 2018 on the biomass, quality, and rhizospheric soil bacterial community of pakchoi in Pb-contaminated soil were further investigated. The results showed that Hao 2018 reduced the Pb concentration in soil filtrate (16%–75%), and its EPS production increased in the presence of Pb^2+^. When compared to the control, Hao 2018 remarkably enhanced pakchoi biomass (10.3%–14.3%), decreased Pb content in edible tissues (14.5%–39.2%) and roots (41.3%–41.9%), and reduced the available Pb content (34.8%–38.1%) in the Pb-contaminated soil. Inoculation with Hao 2018 raised the pH of the soil, the activity of several enzymes (alkaline phosphatase, urease, and dehydrogenase), the nitrogen content (NH_4_^+^-N and NO_3_^−^-N), and the pakchoi quality (Vc and soluble protein content), while also raising the relative abundance of bacteria that promote plant growth and immobilize metals, such as *Streptomyces* and *Sphingomonas*. In conclusion, Hao 2018 reduced the available Pb in soil and pakchoi Pb absorption by increasing the pH and activity of multiple enzymes and regulating microbiome composition in rhizospheric soil.

## 1. Introduction

In recent years, with population growth and industrial development, the use of pesticides and fertilizers and the large-scale exploitation of mineral resources have led to an increase in emissions of heavy metal pollutants, which enter agricultural soil ecosystems through dust and precipitation (Huang et al., 2019; Hossain Bhuiyan et al., 2021; Qin et al., 2021). Most of the heavy metal ions entering the soil are not degraded by microorganisms, but are either enriched in the soil or concentrated by biological action and accumulated in the food chain, ultimately reducing vegetable yields and threatening human health (Nagajyoti et al., 2010; Saadani et al., 2016; Rouillon et al., 2017). Lead (Pb) is one of the main metal elements polluting arable land worldwide (Kushwaha et al., 2018). Vegetables can accumulate heavy metals through root uptake, and excessive Pb accumulation in vegetables offers a significant risk to human health (Pelfrêne et al., 2013). In China, enhanced heavy metal concentrations is an increasing problem associated with rapid industrialization and agricultural intensification, with lead concentrations in agricultural soils of China ranging from 7.6 to 77.27 mg kg^−1^ (Bi et al., 2018; Shifaw, 2018; Huang et al., 2019). Irreversible damage to the human body caused by excessive Pb accumulation can cause respiratory, cardiovascular, neurologic, digestive, and urinary diseases (Boskabady et al., 2018). In order to minimize the accumulation of heavy metals in vegetables, eco-friendly, economical, and energy-efficient procedures are urgently required.

Physical and chemical approaches for remediating soils contaminated with metals have limitations, including the fact that they modify soil microbial communities, which in turn changes soil characteristics, is time-consuming and expensive, and creates secondary pollutants (Tangahu et al., 2011; Yao et al., 2012; Zubair et al., 2016). These methods can only change the form of the problem and cannot completely eliminate the contaminants (Gomes et al., 2016). As a result, the *in situ* application of heavy metal-immobilizing bacteria, a new soil immobilizer, has been widely employed to encourage plant growth and minimize metal absorption of plants by forming indole acetic acid (IAA), siderophores, and reducing metal efficacy in soil (Ma et al., 2011; Wang et al., 2016; Han et al., 2018, 2020; Wang et al., 2020). Bacteria can expel heavy metals through permeable barriers or active export, detoxify them through chemical modification, and physically sequester them with extracellular polymers that bind metals, such as liposaccharides and exopolysaccharides (EPS) (Rouch et al., 1995; Guibaud et al., 2003; Morcillo and Manzanera, 2021).

The absorption and immobilization of heavy metals by EPS are carried out through different mechanisms such as ion exchange, complexation, precipitation etc. (De Philippis et al., 2011; Gupta and Diwan, 2017). Previous studies have suggested that EPS-producing bacteria could effectively reduce the bioavailability of soil metals and alleviate stressed conditions, thereby promoting plant growth and health (Karthik et al., 2017; Mukherjee et al., 2019; Zainab et al., 2020). For example, *B. xiamenensis* PM14 and *Bacillus gibsoni* PM11, two EPS-producing bacteria, improved nutrient availability and development of *Linum usitatissimum* by reducing the stress conditions caused by metals (Zainab et al., 2020). According to Karthik et al. (2017), *Cellulosimicrobium funkei* AR6 produced more EPS when Cr (VI) concentration increased (from 0 to 250 μg/mL), demonstrating the function of EPS as a defensive mechanism for reducing heavy metal stress in rhizospheric soils of plants. Notably, Mukherjee et al. (2019) observed that *Halomonas* sp. Exo1 exopolysaccharide production alleviated heavy metal stress through EPS-mediated biosorption in rice exposed to arsenic (2 mM). In addition, *Pseudomonas. sp.* MHR6, an EPS-producing bacterium, has been shown to decrease the adverse effects of salinity stress on plant growth by enhancing seedling growth, antioxidant levels, the osmotic regulator, and chlorophyll accumulation (Liu et al., 2022).

EPS secretion is an efficient biosorbent material essential for many marine microbes (Baria et al., 2022). Because they have evolved unique protective mechanisms and metabolic pathways to survive in harsh conditions, marine and marine extremophilic bacteria (those that can survive in extreme conditions of temperature, pressure, or the presence of toxic elements like heavy metals) have a greater capacity than other bacteria to produce unique bioactive compounds like EPSs (Casillo et al., 2018; Parrilli et al., 2021). Because of their strong affinity to heavy metals, certain EPSs that are generated by marine bacteria have found widespread application in the treatment of wastewater to remove heavy metals (Siddharth et al., 2021; Baria et al., 2022). As an illustration, the EPS made by *Cyanothece* sp. ATCC 51142 is particularly efficient in removing several metals from industrial wastes (Shah et al., 2000). Despite the fact that rhizobacterial EPS has the potential to reduce the negative effects of heavy metal stress on plants, very little is known about the impact of heavy metals on the composition of EPS and, as a result, the effects of this on root colonization by high-yielding EPS-producing marine bacteria and their positive impact on plant growth and health (Nocelli et al., 2016; Morcillo and Manzanera, 2021). The impacts of bacteria that produce EPS on the plant rhizosphere have seldom been studied, including the direct effects of bacteria on roots and indirect effects on the composition of soil bacterial communities, which are susceptible to soil Pb contamination (Chen et al., 2018). *Pseudoalteromonas agarivorans* Hao 2018, a high-yielding EPS-producing marine bacterium, was generated from the microbial membrane that is found on the surface of abalone seedlings, and previous studies have shown that its EPS production exhibits antioxidant activity, hygroscopicity, moisture retention, and excellent removal of Cu^2+^ and Pb^2+^ (Wang et al., 2018; Hao et al., 2019).

Pakchoi (*Brassica chinensis* L.) is extensively cultivated in China and accounts for 19% of its total production of leafy vegetables (Liu et al., 2010). Pakchoi is also commonly cultivated throughout the world and is easily contaminated with heavy metals when planted on farms (Liu et al., 2019). In this investigation, the ability of the high EPS-producing bacteria *P. agarivorans* Hao 2018 to produce EPS and absorb Pb was assessed. Additionally, the impacts of the strains on the development, quality, and Pb uptake of edible pakchoi tissues in soil contaminated with Pb were examined. Finally, the bioavailable Pb, and the relative abundance of bacteria producing EPS, as well as the structure and function of bacterial communities in rhizospheric soils, were also evaluated. The results contributed to a better understanding of the functions of high-yielding EPS bacteria in the soil microbial community and Pb immobilization and offered a theoretical basis for the employment of EPS-producing bacteria in the safe growth of plants in soil contaminated with heavy metals.

## 2. Materials and methods

### 2.1. Bacteria and soil

In this investigation, a marine bacterium known as *Pseudoalteromonas agarivorans* Hao 2018, which is responsible for the production of EPS (NCBI accession number: OP703330), was extracted from the microbial membrane that is found on the abalone seedlings’ surface (Hao 2018). Hao 2018 can produce 2.34 g L^−1^ EPS and exhibits Pb resistance (25 mM). The soil was obtained from the uncontaminated farmland of the Maize Research Institute, Shandong Academy of Agricultural Sciences, China (N36°44′, E117°22′). The soil was a laurel loam, and is classified as a Chromic Cambisol according to the USDA texture classification system. Initial values for pH, organic matter, total N, and total P contents were 7.90, 13.65, 1.41, and 2.42 g kg^−1^, respectively.

### 2.2. EPS production and Pb immobilization capabilities

Soil (2.5 kg) was mixed with 10 l of deionized water (1,4, m:v) and shaken for 48 h (25°C, 150 rpm) before being centrifuged for 15 min at 5,000 rpm. A Millipore filter (pore size: 0.45 μm) was employed to filter the supernatant before it was introduced to a sterile fermentation medium containing yeast extract (4.5 g L^−l^), sea salt (35 g L^−l^), and glucose (30 g L^-l^) at a ratio of 4:1 (v/v). The Chen et al. (2016) approach was utilized to investigate Hao 2018’s capacity to scavenge Pb^2+^ in the water column. Hao 2018 was grown in a sterile liquid medium, Zobell 2216E, that contained yeast extract (1 g L^−l^), peptone (5 g L^−l^), sea salt (35 g L^−l^), and a pH of 8 before being collected, cleaned, and resuspended in sterile deionized water. The bacterial suspension was introduced to culture flasks (*n* = 4) holding 200 mL of sterile mixtures, and then various Pb^2+^ concentrations (0, 25, 50 mg L^−1^) were added to the mixtures; the non-inoculated treatment served as the control.

At 25°C and 180 rpm, all culture flasks were shaken. OD_600_ was assessed to quantify the amount of bacteria present at 0, 24, 48, 72, and 96 h. A pH meter (Leici PHSJ-4F, China) was employed to evaluate the pH values of the samples. After centrifuging the culture medium for 10 min (25°C, 10,000 rpm), the supernatants were taken and the Pb^2+^ concentration was determined utilizing inductively coupled plasma mass spectrometry (ICP-MS, Agilent 7500a, United States). The phenol-sulfuric acid approach was employed to measure polysaccharide formation (Dubois et al., 1956).

### 2.3. Pot test

Treated soil (4.8 kg) with or without artificial Pb^2+^ (0, 25 and 50 mg kg^−1^) supplementation was added to plastic pots (35 cm × 28 cm; height × width) and equilibrated for 45 days. Each treatment was applied in four separate pots at varying concentrations. Surface-sterilized pakchoi (*Brassica chinensis* L.) seeds were planted in each pot, and following germination, the plants were divided into 15 seedlings per pot. The *P. agarivorans* Hao 2018 strain was grown in a seed medium with yeast extract (1 g L^−l^), peptone (5 g L^−l^), sea salt (35 g L^−l^), and a pH of 8 for 10 h (25°C) before being resuspended in sterile water to a concentration of 1 × 10^8^ cells mL^−1^. Using a sterile syringe, bacterial suspension (90 mL) was injected into the vegetable’s root zone at the third leaf stage while sterile water served as the control. The experiment with the pot was conducted in a greenhouse (at 25 ± 3°C, humidity at 70% and photoperiod of 10 or 12 h per day) for 45 days.

### 2.4. Analysis of Pb concentration and biochemical indexes of pakchoi

Pakchoi plants were collected and categorized into edible parts and roots for subsequent analysis. In order to eliminate metals and dust accumulated in the root-free region, the plant biomass (the roots and edible sections) was thoroughly rinsed with distilled water and ethylenediaminetetraacetic acid (EDTA, 0.01 M) right after harvesting. Pakchoi biomass was then dried at 65°C after being deactivated for 30 min at 105°C to achieve a stable dry weight (DW). The oven-dried roots and edible sections of pakchoi were crushed and digested in an acidic mixture to determine the amount of Pb contained (HClO_4_; HNO_3_: at a ratio of 1:2) (Jones and Case, 2018). Following that, ICP-MS was employed to measure the Pb content. The vitamin C (Vc), nitrite content, and soluble protein in fresh pakchoi edible tissues were determined using the standard method (Li et al., 2000).

### 2.5. Determination of Pb concentration, chemical properties, Hao 2018 colonization and enzyme activity in soil

A particular quantity of firmly bound root soils (rhizospheric soils) was collected from each pot, then allowed to air dry before being crushed, and sieved (2 mm). The pH of the soil was determined in suspension (1:2.5 H_2_O) with a pH meter (Leici PHSJ-4F, China) (Zhao et al., 2018). The concentrations of available Pb were acquired by mechanically shaking air-dried sample with DTPA extract (5 mM DTPA, 10 mM CaCl_2_, 100 mM triethanolamine, pH 7.30) (Lindsay and Norvell, 1978). After oscillation, the solution was centrifuged and filtrated using filter membrane (0.45 μm). To measure the DTPA-extractable Pb concentration in soil, the filtrate was performed within 48 h *via* ICP-MS.

Fresh soil samples were kept at −20°C for the measurement of soil nitrate-nitrogen (NO_3_^−^-N), ammonia nitrogen (NH_4_^+^-N), and enzyme activity (dehydrogenase, urease, and alkaline phosphatase). The remaining soil sample was stored (−80°C) for total DNA extraction. Measurements of soil mineral nitrogen (NO_3_^—^N and NH_4_^+^-N) were conducted employing standard automated colorimetric procedures based on the cadmium reduction and the Berthelot reaction approach (Henriksen and Selmer-Olsen, 1970; Rhine et al., 1998). Based on the procedure previously disclosed, the Hao 2018 colonization in the rhizospheric soil was examined (Alami et al., 2000). Briefly, the rhizospheric soil was serially diluted (0.85% KCl) and each dilution’s aliquots were placed on Zobell 2216E solid medium. Mucoid colonies resembling those from Hao 2018 were quantified as CFU after being incubated for 72 h at 30°C. In order to confirm that the mucoid colonies were Hao 2018, about 10% of the isolates that were identical to it were chosen at random, and their genetic fingerprints were identified utilizing rep-PCR with enterobacterial repetitive intergenic consensus (ERIC) sequences as primers.

Determination of soil dehydrogenase activity (SDH) was accomplished by incubating soil samples with triphenyl tetrazolium chloride (TTC) for 24 h at 25°C, and then evaluating the absorbance at 485 nm of the resulting triphenyl formazan (TPF) (Klein et al., 1971). Soil alkaline phosphatase (ALP) activity was evaluated by measuring the quantity of ρ-nitrophenol released (absorbance at 420 nm) (Tabatabai and Bremner, 1969). According to a previously reported approach, urease activity (URE) was measured employing absorbance at 527 nm (Douglas and Bremner, 1971).

### 2.6. Analysis of soil bacterial community

A DNA kit (Omega Biotek, Inc.) was utilized to extract the entire DNA from soil samples. Agarose gel electrophoresis was utilized to evaluate the concentration and purity of the retrieved genomic DNA. The forward primer 338 F (5′- ACTCCTACGGGAGGAGCA −3′) and reverse primer 806 r (5-GGACTACHVGGT-WTCTAAT −3′) were used to amplify the variable region of 16Sr RNA. PCR amplification was conducted according to the method of a previous study (Zhao et al., 2016). A QiaQuick PCR purification kit (QINGEN, Hilden, Germany) was used to purify the PCR products and an Illumina Hiseq 2000 (Illumina Inc., San Diego, United States) was used for high-throughput sequencing. The resulting sequence data were analyzed with the software program Mothur.^1^ The taxonomic units (OTUs) were classified using the UCLUST sequence comparison tool. To estimate the community distribution diversity, the Shannon, Simpson, Chao1 and ACE indices were calculated. Principal coordinates analysis (PCoA) of the microbial community was carried out on the distance matrices, and the coordinates were then used to generate 2D graphical results. Metastas was utilized to compare the abundances of taxa at the genus and phylum levels between groups. To obtain the important indicator taxa, the RandomForest package (ntree = 1,000) was used to conduct Random Forest analyses (Zhang et al., 2018).

### 2.7. Statistical analysis

All experimental data were statistically analyzed using SPSS 20.0 software (SPSS Inc., USA) and represented as means ± standard deviation (SD). The Shapiro–Wilk and Bartlett’s tests were used to evaluate the normality and homogeneity of variance of the original data. A two-way ANOVA followed by Fisher’s least significant difference (LSD) *post hoc* analysis was used to test for significant differences between treatments (*p* < 0.05). Principal component analysis (PCA) was performed using CANOCO 5.0 software (Microcomputer Power Inc., United States).

## 3. Results

### 3.1. Changes in Pb content, bacterial growth, pH, and EPS production in polluted soil filtrate in the presence of Hao 2018

**Figure 1:**
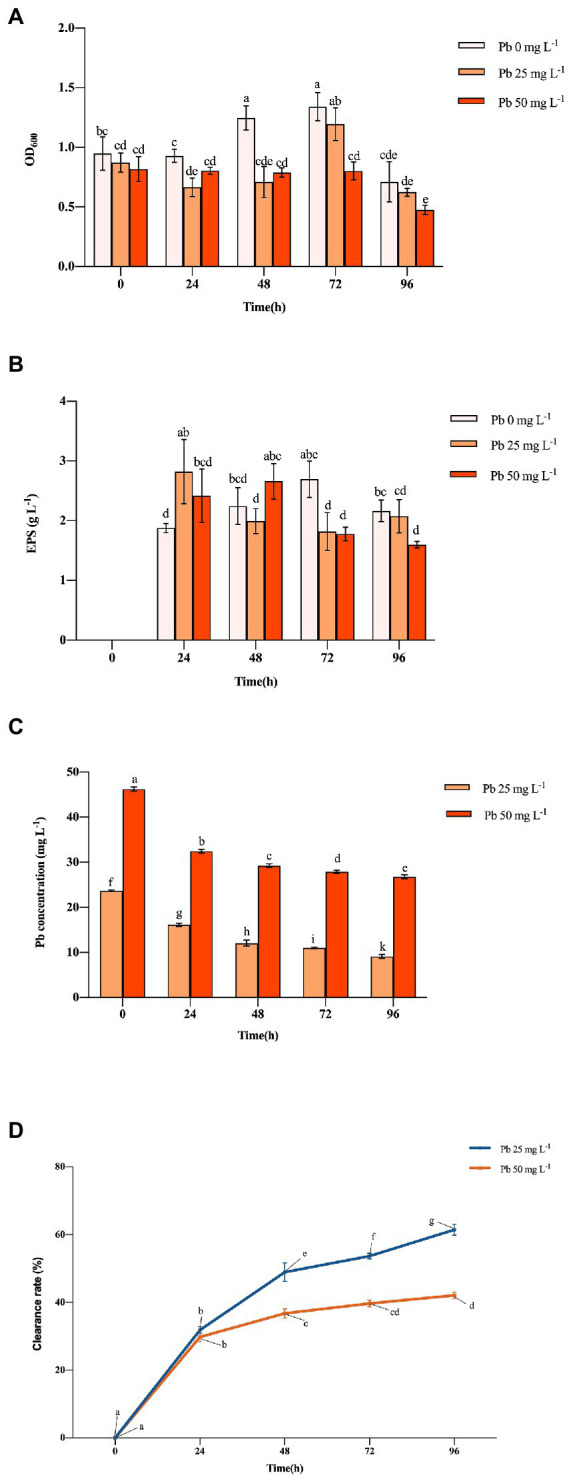
The cell number (indicated by OD_600_) **(A)**, EPS **(B)**, Pb **(C)** content, and clearance rate of Pb **(D)** in the culture solution at different Pb concentrations inoculated with Hao 2018. Each bar represents the mean ± SD (*n* = 4). The same letter indicates that there were no significant differences between all treatments (*p* > 0.05).

The OD_600_ of Hao 2018 generally increased with time, peaking at 1.341 and 1.194 at 72 h under non-Pb and 25 mg L^−1^ Pb^2+^ exposure, respectively (Figure 1A). When the Pb^2+^ content in the solution was 25 mg L^−1^, Hao 2018 had a growth lag period of 48 h. Hao 2018 had a very low growth rate at all time periods when cultured with 50 mg L^−1^ Pb^2+^. Throughout the experiment, the EPS production of Hao 2018 generally increased with time before 72 h, reaching a peak of 2.695, 2.823, and 2.660 g L^−1^ under non-Pb, 25 mg L^−1^ Pb^2+^ and 50 mg L^−1^ Pb^2+^ exposure, respectively (Figure 1A). Its OD_600_ and EPS production decreased over time during 76–96 h for all treatments (Figures 1A,B). The EPS concentration of Hao 2018 increased at 24 and 48 h under Pb^2+^ exposure compared with the control. The concentration of water-soluble Pb decreased significantly as incubation time lengthened (Figure 1C). Under the 25 and 50 mg L^−1^ Pb^2+^ treatments, the Pb clearance rates of Hao 2018 reached their maximum at 96 h (62.52% and 42.10%, respectively) (Figure 1D). In addition, the pH value of Hao 2018 cultures was higher under Pb^2+^ exposure compared with non-Pb exposure, ranging between 5.49 and 6.73 (Suppelmentary Figure S1).

### 3.2. Effect of Hao 2018 on Pb content in rhizospheric soil and Pb uptake in the root and edible tissues of pakchoi

**Figure 2:**
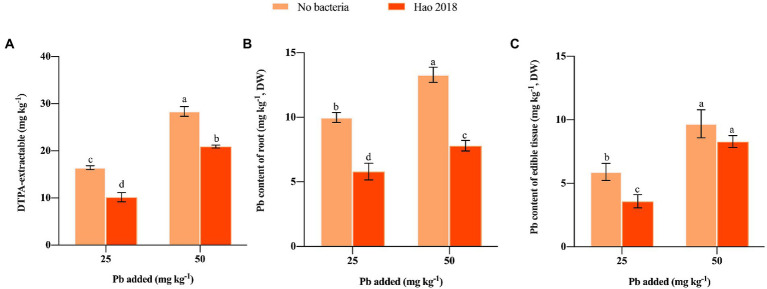
The influence of Hao 2018 on DTPA-extractable Pb content **(A)** Pb content of the root **(B)** and Pb content of the edible tissues **(C)** in the rhizospheric soil of pakchoi. Each bar represents the mean ± SD (*n* = 4). Bars marked with the same letter within each treatment are not significantly different (*p* > 0.05).

Inoculation with Hao 2018 significantly reduced the DTPA-extractable Pb content (34.8–38.1%) in both low and high Pb-contaminated rhizospheric soil compared with the control (Figure 2A). Notably, Hao 2018 dramatically reduced Pb content in the edible tissues (41.3%–41.9%) and roots (14.5%–39.2%) of pakchoi (Figures 2B,C).

### 3.3. Effect of Hao 2018 on pakchoi biomass and quality

**Figure 3:**
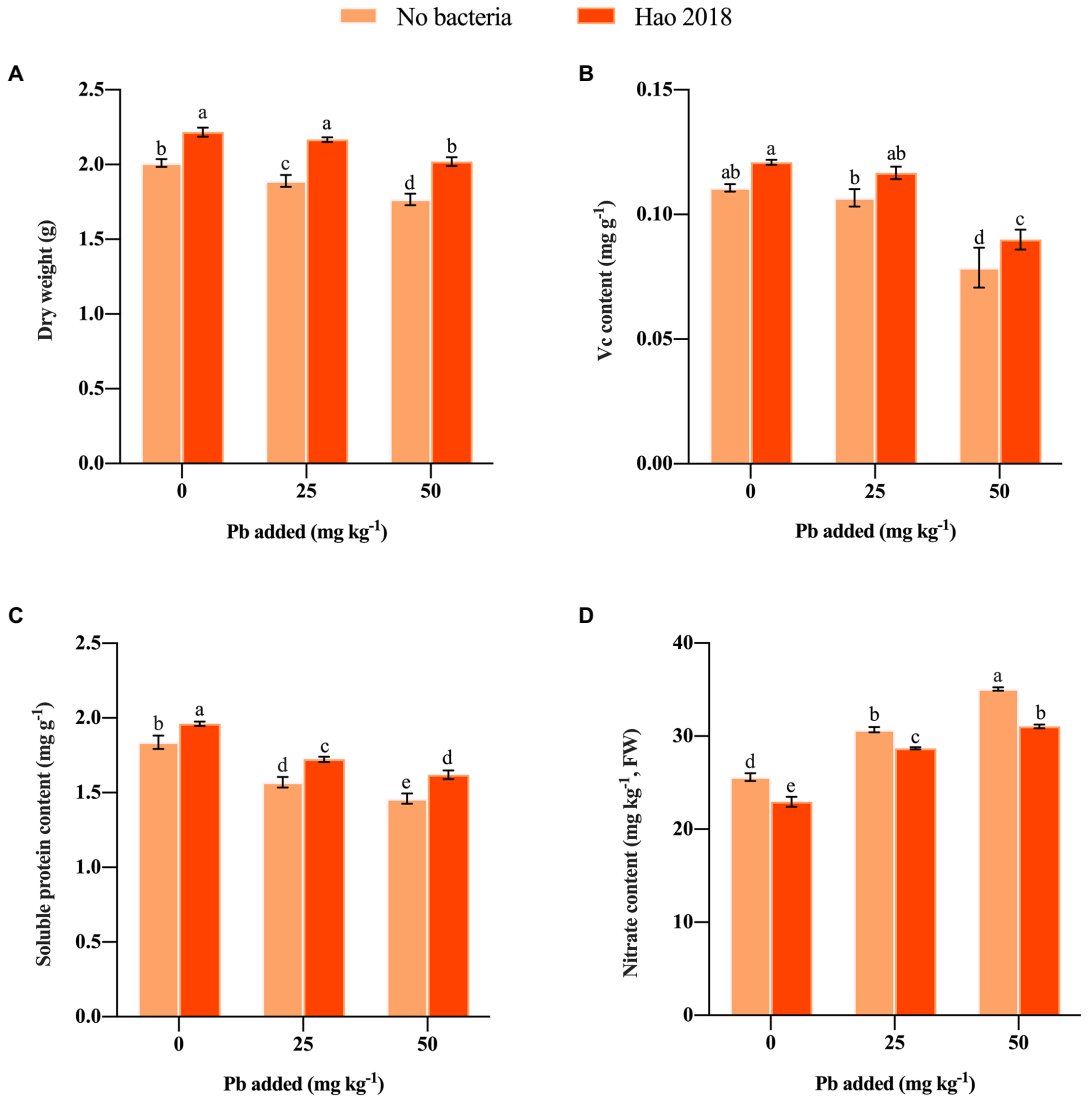
Effect of Hao 2018 on biomass **(A)**, vitamin C (Vc) **(B)**, soluble protein **(C)** and nitrite content **(D)** of the edible tissues of pakchoi. Each bar represents the mean ± SD (*n* = 3). Values followed by the same letter were not significantly different (*p* > 0.05).

After inoculation Hao 2018, pakchoi biomass (10.3%–14.3%) (Figure 3A), Vc contents (9.3%–14.4%) (Figure 3B) and soluble protein contents (6.7%–10.9%) (Figure 3C) were significantly increased. However, pakchoi nitrite content in the Hao 2018-inoculated group was significantly lower (6.5%–11.5%) than that in the non-inoculated group (Figure 3D).

### 3.4. Effect of strain Hao 2018 on soil properties and enzyme activities

**Figure 4:**
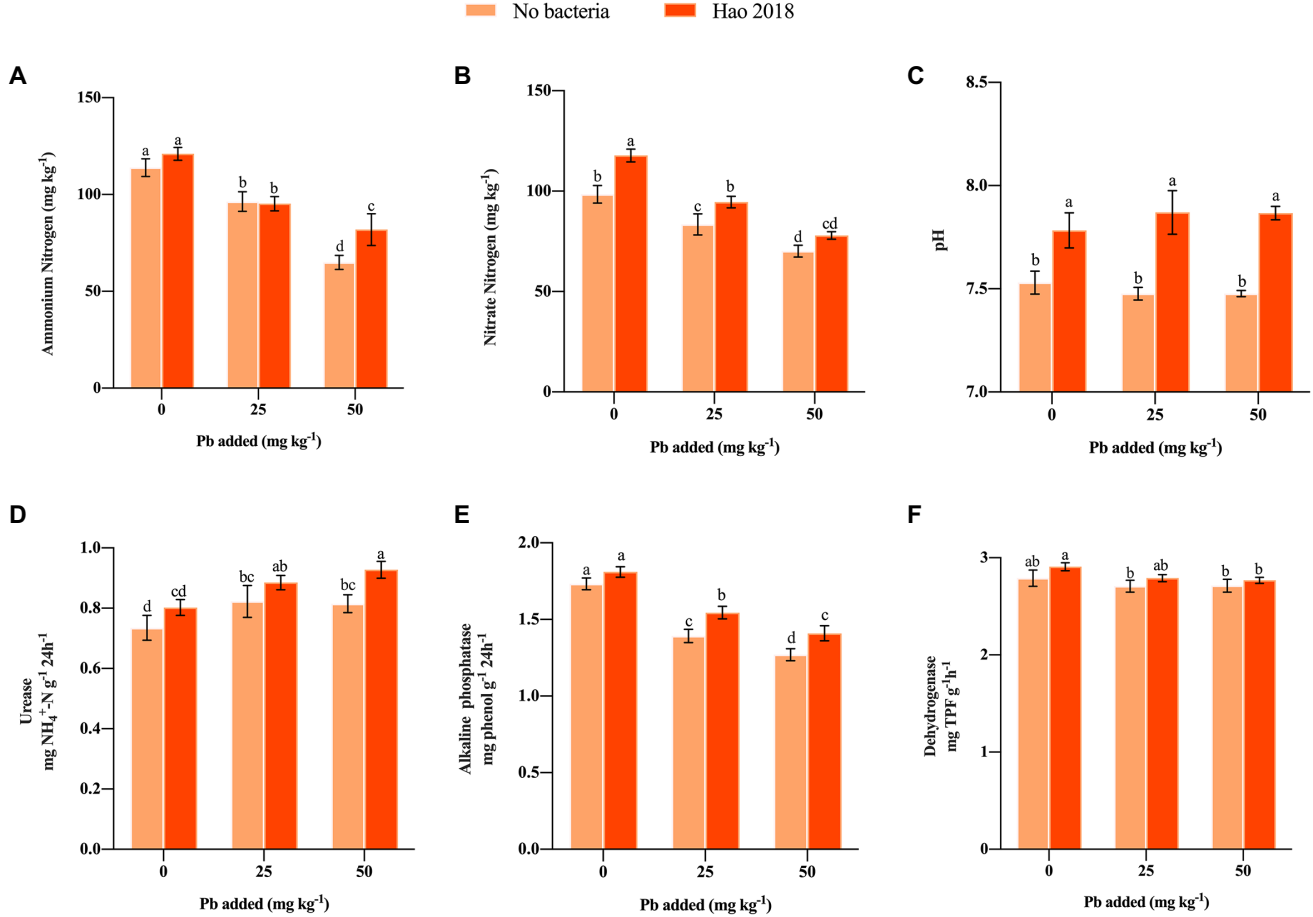
Effect of Hao 2018 on NH_4_^+^-N content **(A)**, NO_3_^−^-N content **(B)** and pH **(C)** in rhizospheric soil. The influence of Hao 2018 on urease **(D)**, alkaline phosphatase **(E)** and dehydrogenase **(F)** activity in rhizospheric soil. Each bar represents the mean ± SD (*n* = 4). Bars marked with the same letter are not significantly different (*p* > 0.05).

NH_4_^+^-N and NO_3_^−^-N content in the rhizospheric soils generally decreased under Pb exposure regardless of Hao 2018 inoculation (Figures 4A,B). However, inoculation with Hao 2018 caused a dramatic increase in NH_4_^+^-N (26.3%) content compared to the control treatment (Figure 4A). Similarly, after inoculating with Hao 2018, the NO_3_^−^-N content increased markedly (11.25–19.7%) compared with the control from the non- and Pb-contaminated (25 mg L^−1^) soils (Figure 4B). The pH (0.2–0.4 units) of rhizospheric soil after inoculation with Hao 2018 was significantly higher than that of the control (Figure 4C). The colonization of Hao 2018 in pakchoi rhizospheric soil was also analyzed. After 45 days of inoculation, the cell number of Hao 2018 was 2.4–3.5 × 10^4^ cfu g^−1^ of fresh soil.

The effect of Hao 2018 on urease, alkaline phosphatase, and dehydrogenase activity in rhizospheric soil was investigated (Figures 4D–F). In the absence of Hao 2018, urease activity in Pb-contaminated soil was higher than that in uncontaminated soils, alkaline phosphatase activity gradually decreased with increasing Pb^2+^ concentration, while dehydrogenase activity did not change significantly as the Pb^2+^ concentration increased. However, urease (7.6%–13.8%), alkaline phosphatase (4.5%–11.0%), and dehydrogenase activity (2.0%–4.2%) of both non- and Pb-polluted treatments generally increased following inoculation with Hao 2018 (Figure 4D).

### 3.5. Principal component analysis

**Figure 5:**
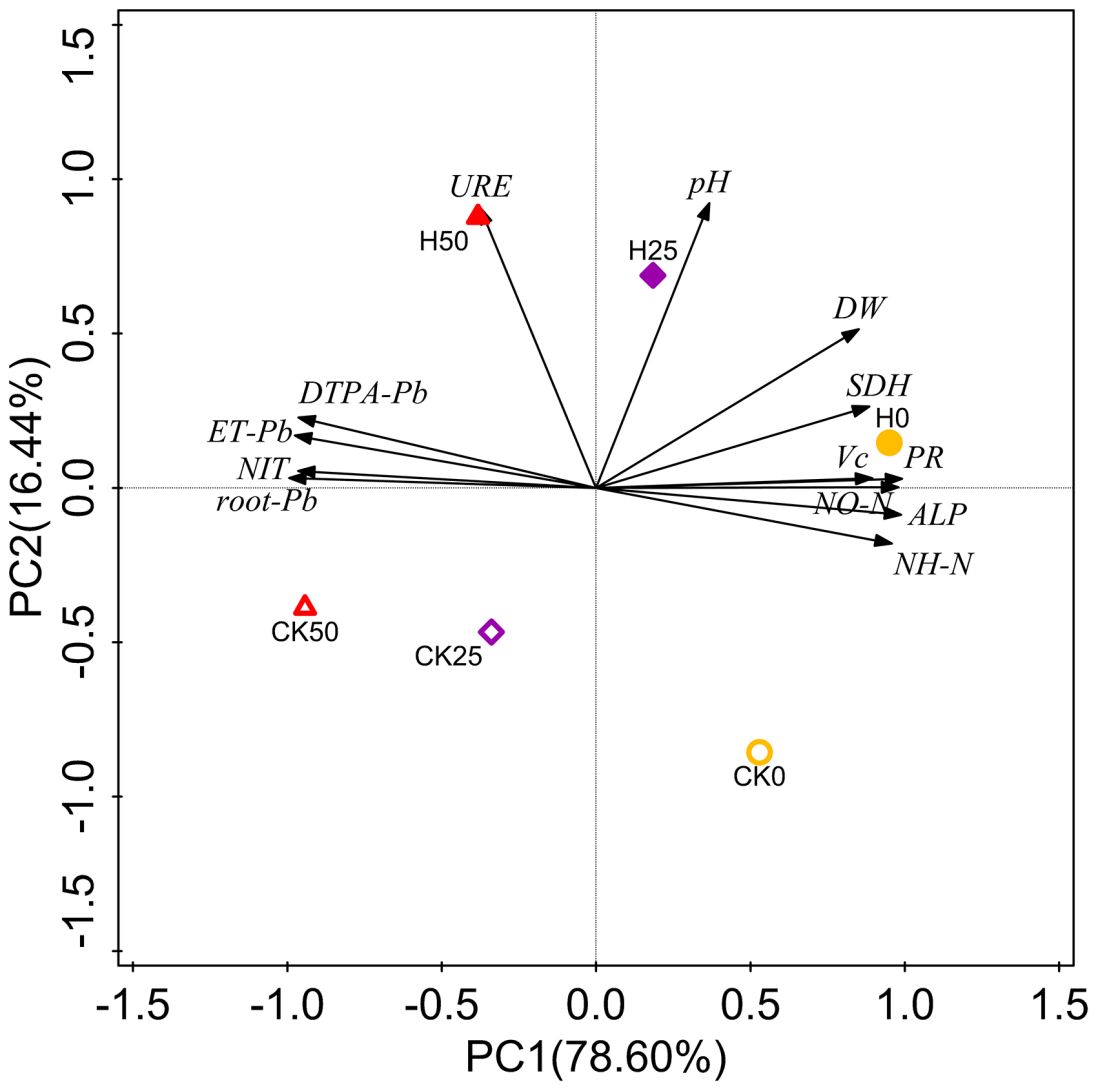
PCA of Pb content in soil and pakchoi, biochemical indices of pakchoi, and the chemical properties and enzyme activity in its rhizospheric soils. DTPA-Pb, DTPA-extractable Pb content; root-Pb, Pb content in roots; ET-Pb, Pb content in edible tissues; DW, dry weight; NIT, nitrite content; VC, vitamin C content; PR, soluble protein content; NH-N, ammonia nitrogen (NH_4_^+^-N) content; NO-N, nitrate-nitrogen (NO_3_^−^-N) content; URE, urease activity; ALP, alkaline phosphatase activity; SDH, dehydrogenase activity.

PCA was applied to all data and revealed 95.04% of the total variance (Figure 5). PC1 represented 78.60% of the variance, highlighting the separation between non- and Pb-contaminated treatments. The second component (explaining 16.44% of the variance) identified the separation of uninoculated treatments and those inoculated with Hao 2018. The degree of Pb contamination was positively correlated with Pb content in soil and pakchoi and negatively correlated with rhizospheric soil chemical properties and enzyme activity, pakchoi biomass, and quality. However, Hao 2018 inoculation treatments were positively correlated with rhizospheric soil chemical properties and enzyme activity, pakchoi biomass, and quality. Hao 2018 inoculation treatments were also negatively correlated with Pb concentration in rhizospheric soil and pakchoi (root and edible tissues).

### 3.6. Bacterial community diversity and structural differences in rhizospheric soil

**Figure 6:**
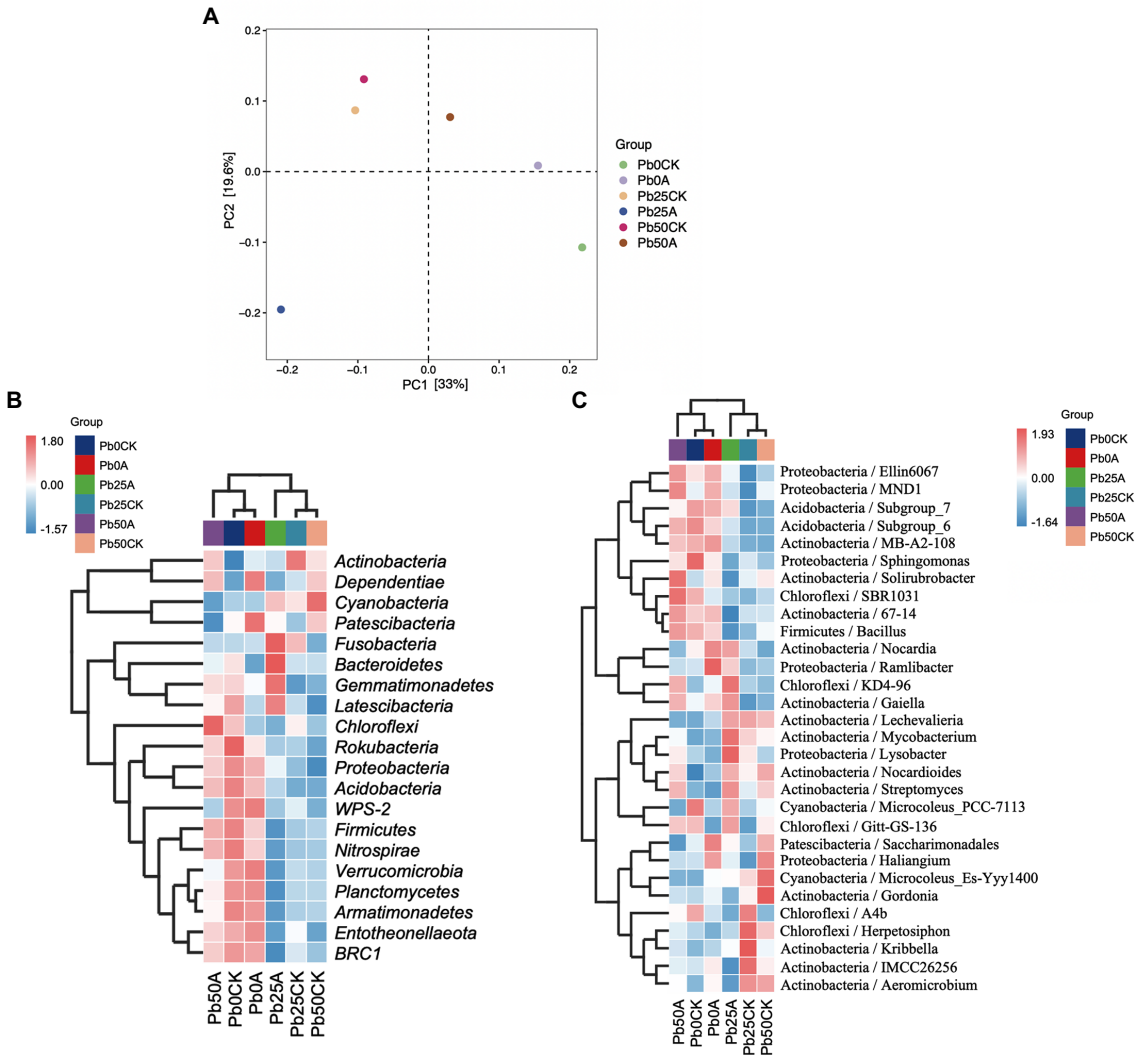
Principle coordinates analysis (PCoA) showed a pattern of inoculation grouping based on the weighted UniFrac distance of all communities. Each colored dot represents a treatment **(A)**. The heatmap of the top 20 phylum **(B)** and top 30 genera **(C)** in the rhizospheric soil of pakchoi with different Pb concentrations. The relative values for each bacterial phylum or genera were depicted by color intensity according to the legend at the top right corner of the figure.

Details of 16S rRNA gene sequencing experiments were presented in Supplementary Table S1. Based on OTUs with 97% similarity, Pb application had no effect on bacterial alpha diversity (Observed species and Chao1, Shannon, and Simpson indices) in rhizospheric soil. In the rhizospheric soil with high (50 mg kg^−1^) Pb, three α-diversity indices (Observed species and Chao1 and Shannon indices) were significantly higher in the Hao 2018 treatment than those in the control. However, bacterial α-diversity was not significantly different in rhizospheric soils with non- and Pb contamination (25 and 50 mg kg^−1^) in the presence of Hao 2018 (Supplementary Table S2). To better observe the changes in the composition of the inter-rhizospheric soil bacterial community caused by the inoculated strain Hao 2018, PCoA analysis was performed and the principal components 1 and 2 explained 33 and 19.6% of the total variations, respectively (Figure 6A). The results clearly showed that inoculation with strain Hao 2018 changed bacterial community composition. The rhizospheric soil communities inoculated with Hao 2018 were grouped together and significantly separated from those of the uninoculated control.

**Figure 7:**
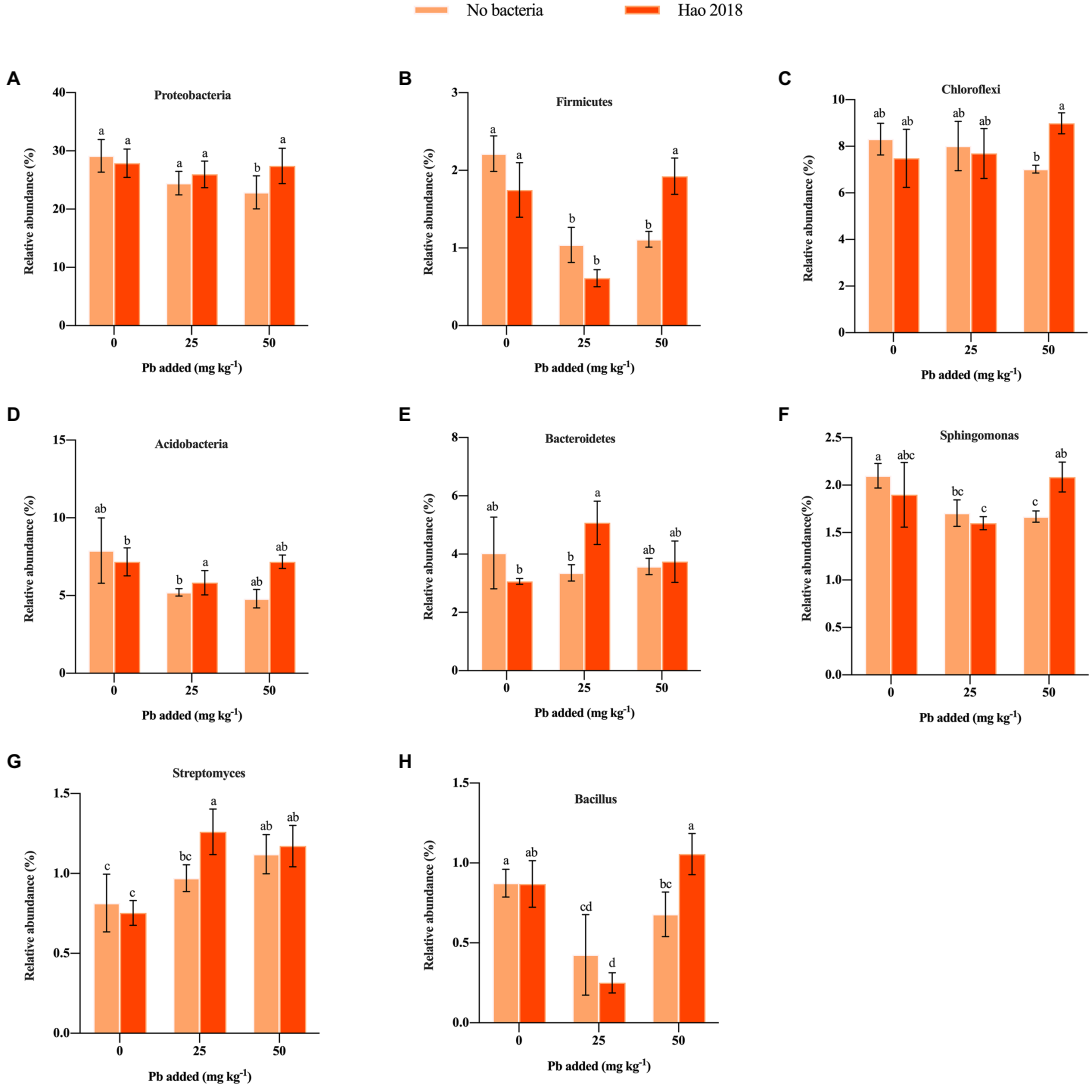
The influence of Hao 2018 on the relative abundances of the phyla *Proteobacteria* **(A)**, *Firmicutes* **(B)**, *Chloroflexi* **(C)**, *Acidobacteria* **(D)**, and *Bacteroidetes* **(E)**, and the genera *Sphingomonas* **(F)**, *Streptomyces* **(G)**, and *Bacillus* **(H)** in rhizospheric soil. Each bar represents the mean ± SD (*n* = 4). The same letter indicates no significant differences between the groups (*p* > 0.05).

A total of 1,254,703 high-quality sequences belonging to 45 phyla were obtained. *Actinobacteria* (30.3%–38.4%) was the dominant phylum, followed by *Proteobacteria* (22.9%–29%), *Cyanobacteria* (10.4%–17.8%), *Chloroflexi* (7.4%–9%), *Acidobacteria* (5.2%–7.9%) and *Bacteroidetes* (3%–5.1%) (Supplementary Figure S2). Figure 6B shows the top 20 phyla, such as *Proteobacteria*, *Firmicutes*, *Chloroflexi*, *Acidobacteria*, and *Bacteroidetes* in the rhizospheric soil of pakchoi with different Pb concentrations. In this study, when Hao 2018 was not inoculated, the relative abundance of *Proteobacteria*, *Firmicutes*, *Chloroflexi*, *Acidobacteria*, *Bacteroidetes, BRC1*, *Entotheonellaeota*, *Nitrospirae*, and *Gemmatimonadetes* was lower in soils supplemented with Pb (25, 50 mg L^−1^) than in soils without Pb contamination (Figures 6B, 7A–E). When the Pb concentration was 50 mg L^−1^, the abundance of the phyla *Proteobacteria*, *Firmicutes*, *Chloroflexi*, and *Acidobacteria* in the soil inoculated with Hao 2018 was higher than that in the uninoculated soil (Figures 6B, 7A–D). In the 25 mg kg^−1^ Pb contaminated soil, the relative abundance of *Acidobacteria* and *Bacteroidetes* in the soil inoculated with Hao 2018 was significantly increased compared with the uninoculated control. Additionally, the relative abundances of the phyla *BRC1*, *Entotheonellaeota*, *Nitrospirae*, and *Gemmatimonadetes* in high Pb-polluted soil were remarkably higher than those of other treatments in the presence of Hao 2018 (Figure 6B). Similar results were observed for the genera *Sphingomonas* and *Bacillus*, with the relative abundances of these genera in soils inoculated with Hao 2018 being significantly higher than those in uninoculated controls under high Pb-polluted conditions (Figures 6C, 7F,H). In the presence of 25 mg kg^−1^ Pb, a significantly higher relative abundance of *Streptomyces* was found in rhizospheric soils inoculated with Hao 2018 compared to the uninoculated control (Figures 6C, 7G). The genera *Nocardioides, Lysobacter*, *Gaiella*, and *Solirubrobacter* also had high relative abundance in the presence of Hao 2018 in non- and Pb-contaminated rhizospheric soils (Figure 6C).

## 4. Discussion

Reducing the bioavailability or mobility of heavy metal ions is very important to reduce the harm caused by their absorption and accumulation by vegetables (Khan et al., 2015; He et al., 2019). Microbial immobilization of heavy metals in soil is an economical and environmentally friendly way to reduce plant absorption of heavy metals (Ma et al., 2011; Han et al., 2020). Here, we explored the effects of the EPS-producing bacterium Hao 2018 on the immobilization of Pb in soil filtrate, plant growth, and Pb accumulation in pakchoi. The results of this study suggest that Hao 2018 effectively reduced the concentration of Pb (16–75%) in soil filtrate, and its EPS production increased in the presence of Pb^2+^. Additionally, EPS-producing bacteria Hao 2018 affected Pb bioavailability in rhizospheric soil, the results showed that strain Hao 2018 could effectively reduce the content of DTPA-extractable Pb in the rhizospheric soil, resulting in the reduction of Pb^2+^ absorption by the roots and edible tissues of pakchoi. Furthermore, strain Hao 2018 could effectively increase the biomass, Vc, and soluble protein content of pakchoi (Figure 3). Studies have shown that high concentrations of Pb^2+^ can affect the rooting and germination of seeds, thereby inhibiting photosynthesis and mineral absorption of the aboveground parts (Shen et al., 2016). Therefore, inoculation with Hao 2018, which is highly effective at immobilizing Pb^2+^ in soil, had a positive effect on the growth and development of pakchoi.

Nitrogen is an essential macronutrient that is often a limiting factor for normal plant growth (Giansoldati et al., 2012). Plants mainly absorb N in two forms, nitrate (NO_3_^−^) and ammonium (NH_4_^+^), which can be easily absorbed, thus increasing their biomass (Taiz et al., 2015). Similarly, Hao 2018 inoculation improved the biomass and quality of pakchoi in the presence of Pb pollution, which may be related to the increase in NH_4_^+^-N and NO_3_^−^-N content in rhizospheric soil. Previous studies have also found that the As-tolerant strain *Halomonas* sp. Exo1 promoted rice growth under metal As stress primarily through EPS secretion (Mukherjee et al., 2019).

Nitrogen (N) management is an agronomic strategy designed to prevent crops against metal contamination (Luo et al., 2012; Mao et al., 2014). Some scholars believe that NO_3_^−^ absorption by plants is accompanied by proton (H^+^) absorption, leading to an increase in rhizosphere pH (Thomson et al., 1993). The basic function of the nitrate transporter (NRTs) gene is NO_3_^−^ absorption. Zhu et al. (2019) found that Pb^2+^ induced the expression of the NRT1.1 gene and its encoded protein under co-supply of NO_3_^−^ and NH_4_^+^, thereby promoting NO_3_^−^ uptake, increasing rhizosphere pH, and reducing Pb availability, and thus reducing Pb uptake in *Arabidopsis*. Many studies have suggested that the inoculation of soil with heavy metal-immobilizing bacteria increases soil pH and thereby reduces the availability of heavy metals (Madhaiyan et al., 2007). According to our study, the EPS-producing bacterium Hao 2018 contributes to a substantial increase in the supply of NH_4_^+^ and NO_3_^−^ to the rhizospheric soil and its pH, thereby reducing the level of DTPA-extractable Pb in the rhizospheric soil and the uptake of Pb by pakchoi.

The activity of soil enzymes is an essential measure of the health of soil ecosystems, especially those that have been affected by heavy metal pollution (Masto et al., 2009). Urease and alkaline phosphatase activities in soil are linked to the conversion of soil nitrogen and phosphatase, whereas dehydrogenase activity are crucial to soil respiration. Pb pollution disturbed the biochemical balance of soils in our study as evidenced by the inhibition of soil dehydrogenases (SDH) and alkaline phosphatase (ALP) activity and the stimulation of soil urease (URE) activity. Previous studies have also reported complex effects of heavy metals on soil enzyme activity (Khan et al., 2010; Pan and Yu, 2011; Wang et al., 2020). The increased enzyme activity in Hao 2018 inoculated-rhizospheric soil might imply that it may contribute to improving the soil biochemical reaction and thus improve plant growth. Similar to our research, Wang et al. (2020) found that enzyme (ALP, URE and SDH) activity and plant biomass significantly increased in Cd-contaminated rhizospheric soils inoculated with the γ-PGA-producing bacteria *Bacillus amyloliquefaciens* W25 and *Bacillus subtilis* W7. PCA analysis further verified the positive effect of Hao 2018 on physical and biochemical responses, rhizospheric soil chemical properties, and the enzyme activity of pakchoi under non- and Pb contamination.

Rhizospheric bacteria can alter metal bioavailability by modifying the pH of the surrounding environment, creating chelating agents, and altering redox potentials, which increases the efficiency of microbial bioremediation (Giller et al., 1998). In this study, the composition of the rhizosphere microbiome was affected not only by metal contamination but also by soil microbial modification, and metal contamination resulted in a significant decrease in microbial diversity. Furthermore, the results showed that the α-diversity of the Hao 2018-inoculated metal-contaminated rhizospheric soil was significantly higher than that of the control under high Pb pollution (Supplementary Table S2), indicating the recovery of microbial communities after Hao 2018 inoculation and the effectiveness of the remediation measures we adopted. Previous studies have also found that bacterial phyla including *Proteobacteria*, *Chloroflexi*, *Acidobacteria*, and *Bacteroidetes* have consistently been found to dominate metal-contaminated soils, most likely because of their high tolerance to metals (Jiang et al., 2016; Chen et al., 2019). These microbial communities colonize the rhizosphere due to their functional properties and effects on plant nutrient uptake, growth, and disease suppression (Yadav et al., 2020). The three phyla with the highest abundance in natural soils are *Proteobacteria, Bacteroidetes, and Acidobacteria* (Roesch et al., 2007), and they are believed to be linked to soil carbon and/or nitrogen cycles (Tang et al., 2011; Rawat et al., 2012; Chen et al., 2018). Researchers further point out that the bacterial genera belonging to *Proteobacteria* and some *Firmicutes* are most capable of dissolving inorganic forms of phosphate into effective forms (Selvakumar et al., 2012; Rana et al., 2019). The results of this study showed that Pb contamination reduced the abundance of *Proteobacteria*, *Firmicutes*, *Chloroflexi*, *Acidobacteria*, and *Bacteroidetes* in rhizospheric soils. Similar to our results, previous studies have found that metal contamination reduced the abundance of *Proteobacteria*, *Chloroflexi*, and *Bacteroidetes* in soil (Berg et al., 2012; Narendrula-Kotha and Nkongolo, 2017). We also observed a general increase in the abundance of *Proteobacteria*, *Firmicutes*, *Chloroflexi*, *Acidobacteria*, and *Bacteroidetes* in the rhizospheric soils inoculated with Hao 2018 compared with control soils under metal-contaminated conditions (especially at 50 mg L^−1^ Pb^2+^) (Figures 6B, 7A–E), which consistently indicate a reduction in metal toxicity in the rhizosphere of pakchoi, as discussed previously. These results suggest that soil inoculated with Hao 2018 caused changes in microbial communities in metal-contaminated soils, making them more similar to uncontaminated soils. When soils with high Pb contamination were inoculated with Hao 2018, the relative abundances of the phyla *BRC1*, *Entotheonellaeota*, *Nitrospirae*, and *Gemmatimonadetes* increased dramatically (Figure 6B). Heavy metal resistance has been attributed to these phyla because of their role in C/N cycling activities such as urea hydrolysis, denitrification, nitrification, calcite precipitation, and EPS production (Zhang et al., 2021).

More importantly, the relative abundance of *Streptomyces, Sphingomonas*, *Nocardioides*, *Bacillus*, *Gaiella*, and *Solirubrobacter* was high in the Pb-contaminated rhizospheric soils inoculated with Hao 2018. Members of those genera were usually found to be metal-tolerant species and/or plant growth-promoting bacteria (PGPB) (Tangaromsuk et al., 2002; Dimkpa et al., 2008; Rodríguez-Tirado et al., 2012; Khan et al., 2014; Mahbub et al., 2016; Tan et al., 2017; Kou et al., 2018; Wang et al., 2020; Geng et al., 2022). In particular, the genera *Sphingomonas*, *Streptomyces*, and *Bacillus* are both metal-immobilizing and plant growth-promoting (So et al., 2001; Tangaromsuk et al., 2002; Dimkpa et al., 2008; Kumar et al., 2012; Rodríguez-Tirado et al., 2012; Khan et al., 2014; Tan et al., 2017). In addition, the presence of Hao 2018 might be linked to an increase in antibiotic-producing microorganisms in plant roots, and an improved plant immune system as a result of Lysobacter enrichment (Puopolo et al., 2016). Moreover, Guo et al. (2020) found that soil alkaline phosphatase activity (ALP) was significantly correlated with the relative abundance of *Lysobacter*, suggesting that this genus may be involved in the response of soil alkaline phosphatase to Pb addition. These findings show the feasibility of employing this EPS-producing bacterium in soil metal remediation by demonstrating an increase in microbial diversity, a reduction in metal bioavailability, and a reorganization of microbial composition in rhizospheric soil following inoculation of Hao 2018.

## 5. Conclusion

This research demonstrated that the EPS-producing bacteria Hao 2018 immobilized Pb by increasing its production of EPS and raising the pH of soil filtrate. Hao 2018 inoculation reduced Pb content in rhizospheric soil and Pb accumulation in pakchoi and increased its biomass, Vc, and soluble protein content. It is worth noting that the Pb content of the edible tissue of pakchoi inoculated with strain Hao 2018 reached the permissible limits of leafy vegetables stipulated by FAO/WHO (2011). Based on the results of this study, it is clear that Hao 2018 has the potential to decrease soil Pb availability and alleviate Pb stress by elevating soil pH, soil enzyme (ALP, URE, and SDH) activity, nitrogen content (NH_4_^+^-N and NO_3_^−^-N), and the relative abundance of bacteria that promote metal immobilization and plant growth, such as *Streptomyces* and *Sphingomonas*. The results of the study highlighted that the EPS-producing bacteria Hao 2018 has great potential for microbial application in remediating Pb-contaminated soils and mitigating heavy metal content in plants. Further research including field trials at metal-contaminated sites is needed to directly assess the *in situ* remediation capability of Hao 2018.

## Data availability statement

The data presented in the study are deposited in the National Center for Biotechnology Information (NCBI) BioProject repository, accession number PRJNA906320.

## Author contributions

RC and YiZ conducted the whole experiment, did the analysis, and wrote the manuscript. RC and LH designed the study and revised the manuscript. LH provided the research grants for this study and also supervised the work. YJ, WeiW, YaZ, NL, GZ, and XW participated in part of the experiment. XX, CD, YL, HY, KS, CH, WeiyW, LZ, and CJ contributed to the data analysis. All authors contributed to the article and approved the submitted version.

## Funding

This research was supported by the Natural Science Foundation of Shandong Province (ZR2022MD097), Education and Industry Integration Innovation Pilot Project of Qilu University of Technology (Shandong Academy of Sciences) (2022JBZ01-06), the Foundation of State Key Laboratory of Biobased Material and Green Papermaking (No. ZZ20190302), the Foundation of Shandong Provincial Key Laboratory of Biosensors (SWCG 2018–01), the Natural Science Foundation of Shandong Province (ZR2012CM019), and the Foundation (No. 202002) of Qilu University of Technology of Cultivating Subject for Biology and Biochemistry, Science, Education and Industry Integration Innovation Pilot Project of Qilu University of Technology (Shandong Academy of Sciences) (2020KJC-ZD08).

## Conflict of interest

The authors declare that the research was conducted in the absence of any commercial or financial relationships that could be construed as a potential conflict of interest.

## Publisher’s note

All claims expressed in this article are solely those of the authors and do not necessarily represent those of their affiliated organizations, or those of the publisher, the editors and the reviewers. Any product that may be evaluated in this article, or claim that may be made by its manufacturer, is not guaranteed or endorsed by the publisher.

## Footnotes

^1^ http://www.mothur.org
