# Supplementary material for: Exopolysaccharide-producing bacteria enhanced Pb immobilization and influenced the microbiome composition in rhizosphere soil of pakchoi (Brassica chinensis L.)
Source: Front Microbiol. 2023 Mar 9;14:1117312. doi: 10.3389/fmicb.2023.1117312 (PMC10034174; doi:10.3389/fmicb.2023.1117312)
Supplement: Supplementary file 1 [file Table_1.DOCX]

**Supplementary Table 1**. Details of 16S rRNA gene sequencing experiments. Raw PE: Number of raw reads; Qualified: Number of high-qualified reads; CK: non-contaminated controls; A: inoculation with *Pseudoalteromonas agarivorans* Hao 2018. Samples are marked as soil Pb contamination level - inoculation treatment - replicate number, e.g. Pb25CK-1 samples collected from the rhizosphere soil of pakchoi, inoculation with strain Hao 2018, non-contaminated with Pb, first replication.

| Treatment | Raw PE（no.） | Qualified（no.） |
| --- | --- | --- |
| Pb0CK-1 | 131948 | 81095 |
| Pb0CK-2 | 118309 | 61608 |
| Pb0CK-3 | 126493 | 69484 |
| Pb0CK-4 | 129797 | 87466 |
| Pb0A-1 | 139073 | 82793 |
| Pb0A-2 | 151032 | 80818 |
| Pb0A-3 | 127629 | 64821 |
| Pb0A-4 | 148818 | 64616 |
| Pb25CK-1 | 136723 | 87648 |
| Pb25CK-2 | 129863 | 74599 |
| Pb25CK-3 | 126683 | 75559 |
| Pb25CK-4 | 146233 | 57391 |
| Pb25A-1 | 122495 | 71358 |
| Pb25A-2 | 122309 | 60299 |
| Pb25A-3 | 108079 | 51040 |
| Pb25A-4 | 191795 | 86597 |
| Pb50CK-1 | 133478 | 72788 |
| Pb50CK-2 | 126592 | 74589 |
| Pb50CK-3 | 122184 | 58901 |
| Pb50CK-4 | 148932 | 66457 |
| Pb50A-1 | 140757 | 90134 |
| Pb50A-2 | 133256 | 79661 |
| Pb50A-3 | 125104 | 68914 |
| Pb50A-4 | 151648 | 64834 |
