# Supplementary material for: Exopolysaccharide-producing bacteria enhanced Pb immobilization and influenced the microbiome composition in rhizosphere soil of pakchoi (Brassica chinensis L.)
Source: Front Microbiol. 2023 Mar 9;14:1117312. doi: 10.3389/fmicb.2023.1117312 (PMC10034174; doi:10.3389/fmicb.2023.1117312)
Supplement: Supplementary file 2 [file Table_2.DOCX]

**Supplementary Table 2.** The influence of strains Hao 2018 on the α-diversity indices of the bacterial communities in the rhizosphere soils of pakchoi with different Pb concentrations. Means of four replicates per treatment are presented with standard deviation. Data followed by the same letters (a-c) within the same line are not significantly different (P > 0.05) according to Tukey’s test.

| Pb added (mg kg^-1^) | Observed species | chao1 | Shannon | Simpson |
| --- | --- | --- | --- | --- |
| **0**^*^ |  |  |  |  |
| No bacteria | 6075±342ab | 6966±263ab | 10.74±0.4ab | 0.991±0.0042b |
| Hao 2018 | 6513±530a | 7791±746a | 11.03±0.2ab | 0.998±0.0005a |
| **25**^*^ |  |  |  |  |
| No bacteria | 5827±736ab | 6978±681ab | 10.70±0.3ab | 0.997±0.0008a |
| Hao 2018 | 7343±1226ab | 7494±1994a | 10.79±0.4ab | 0.997±0.0019a |
| **50**^*^ |  |  |  |  |
| No bacteria | 4903±78b | 5337±267b | 10.20±0.4b | 0.996±0.0023ab |
| Hao 2018 | 6414±470a | 7645±576a | 11.27±0.2a | 0.997±0.0008b |
