# Supplementary material for: Exopolysaccharide-producing bacteria enhanced Pb immobilization and influenced the microbiome composition in rhizosphere soil of pakchoi (Brassica chinensis L.)
Source: Front Microbiol. 2023 Mar 9;14:1117312. doi: 10.3389/fmicb.2023.1117312 (PMC10034174; doi:10.3389/fmicb.2023.1117312)
Supplement: Supplementary file 3 [file Image_1.pdf]

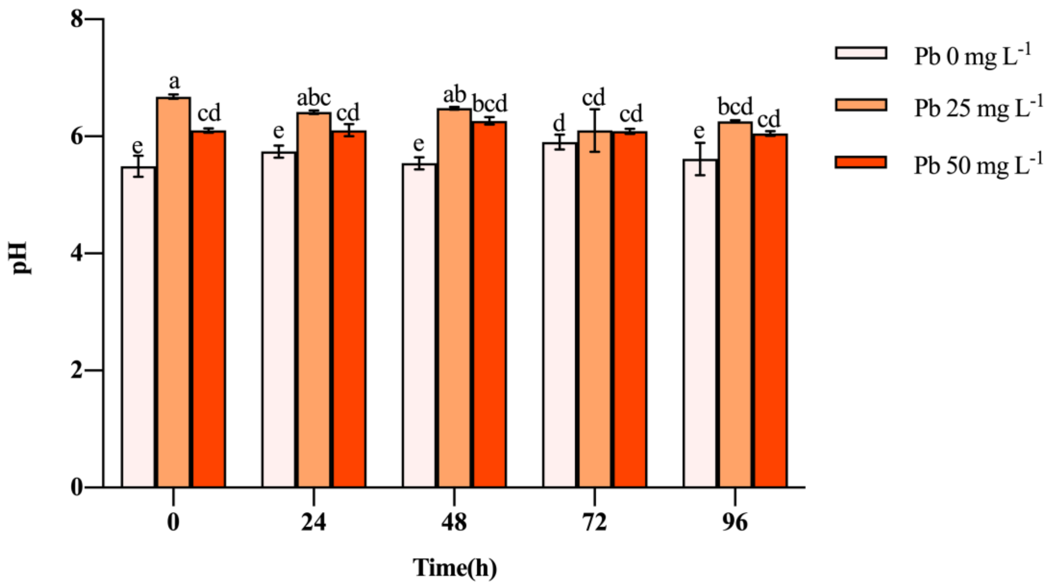

Supplementary Figure 1. The pH in the culture solution at different Pb concentrations inoculated with strains Hao 2018. Error bars are ± standard error (n = 4).
