# Supplementary material for: Exopolysaccharide-producing bacteria enhanced Pb immobilization and influenced the microbiome composition in rhizosphere soil of pakchoi (Brassica chinensis L.)
Source: Front Microbiol. 2023 Mar 9;14:1117312. doi: 10.3389/fmicb.2023.1117312 (PMC10034174; doi:10.3389/fmicb.2023.1117312)
Supplement: Supplementary file 4 [file Image_2.pdf]

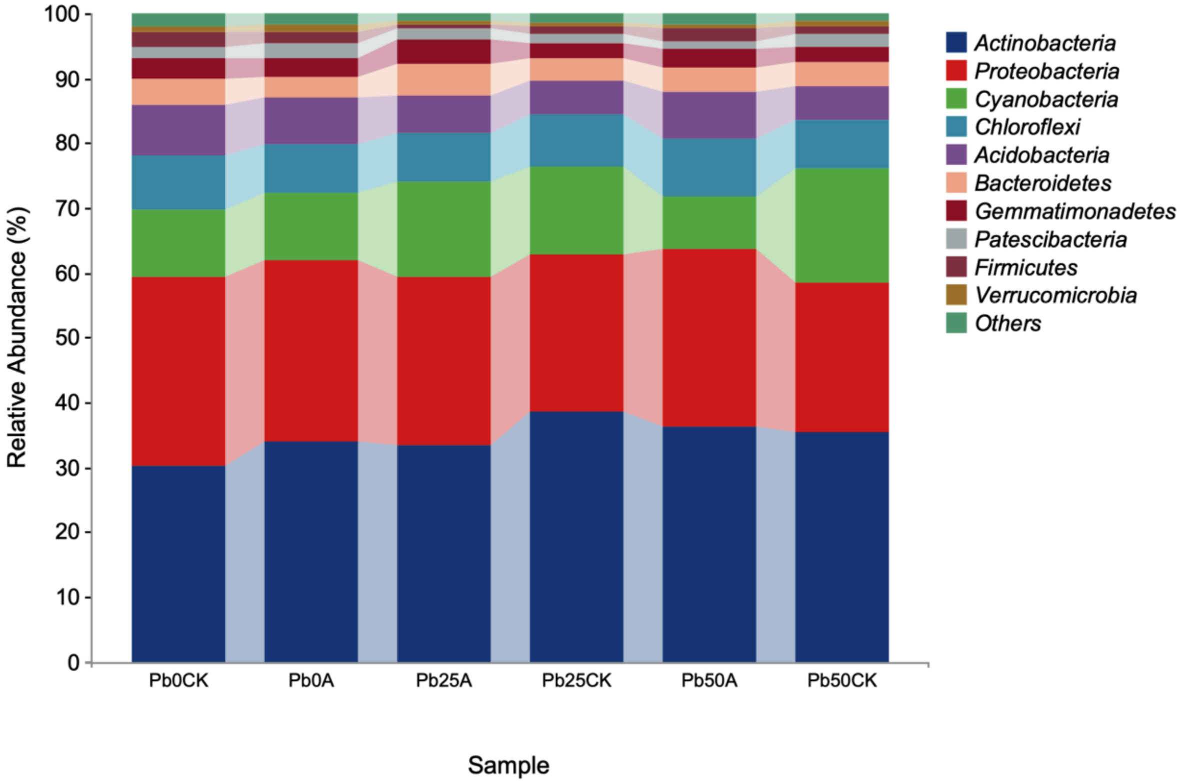

Supplementary Figure 2. The influence of strains Hao 2018 on the relative abundances of bacterial communities at phylum level in the rhizosphere soil of pakchoi with different Pb concentrations.
